# A chopper amplifier with adaptive biasing OTA for biomedical applications, featuring high gain and CMRR

**DOI:** 10.1371/journal.pone.0313423

**Published:** 2024-11-13

**Authors:** Bhaskara Rao Kasipogula, Gurumurthy Komanapalli

**Affiliations:** School of Electronics Engineering, VIT-AP University, Vijayawada, India; Public Library of Science, UNITED KINGDOM OF GREAT BRITAIN AND NORTHERN IRELAND

## Abstract

This paper presents a design of fully differential chopper amplifier employing the flipped voltage follower (FVF) adaptive biasing technique, focusing on its potential use in biopotential recording applications. The suggested architectural OTA incorporates self-cascoded current mirrors (SCCMs) as the active load to achieve a substantial output swing. The FVFs based adaptive biasing approach for the differential input stage boosts extra current and enhances gain and dynamic characteristics. The chopper amplifier attains a common mode rejection ratio (CMRR) of more than 100 dB through the strategic utilization of chopper modulators and pseudo-resistors. Additionally, this device exhibits characteristics such as accurate and stable gain, high input impedance, and a compact physical footprint. The present study also includes a comparison between the suggested structure and the bio-potential amplifiers discussed in the existing literature. This comparison is based on key metrics such as gain, input referred noise (IRN), CMRR, and input impedance (*Z*_*in*_). The proposed structure yielded a gain of 63.72 dB, an IRN of 0.07*nV*_*rms*_, a CMRR of 127.97 dB and a *Z*_*in*_ of 1.54 *G*Ω. The bio-potential chopper amplifier under consideration was constructed and simulations were performed by utilizing the Cadence Virtuoso Spectre simulator tool at 180 nm CMOS technology node.

## Introduction

A signal refers to a tangible occurrence that conveys data or knowledge. The utilization of bio-electrical signals can facilitate the acquisition of data about a biological system being investigated [1]. Neural signals exhibit a diverse range of amplitudes, spanning from tens of micro-volts to several milli-volts. These signals also possess distinct bandwidths, which are shown in Table 1. In this particular scenario, a crucial component of the brain recording system is an appropriate amplifier capable of effectively amplifying low-frequency and low-amplitude signals. It must also effectively counteract the substantial random DC offset, reaching several hundred millivolts, that arises at the interface between the electrode and tissue.

**Table 1:** A summary of various neural signals and recording methods [3, 5, 6].

Classification	Acquisition	Frequency Range (Hz)	Dynamic Range (V)	Comments
Electroencephalogram(EEG)	Surface Electrodes	0.5–100	2–100 *μ*	Multi-Channel (6–32) Scalp Potential
Electrocardiogram (ECG)	Surface Electrodes	0.05–100	1–10 m	Cardiac Action Potential to the surface
Electro-oculogram (EOG)	Surface electrodes	dc–100	10 *μ*–5 m	Steady-corneal-retinal potential
Electroneurogram (ENG)	Needle electrode	100–1 k	5 *μ*–10 m	Potential of a nerve bundle
Action potential (AP)	Micro electrodes	100–2 k	10 *μ*–100 m	Invasive measurement of cell membrane potential
Electroretinogram (ERG)	Micro electrode	0.2–200	0.5 *μ*–1 m	Evoked flash potential
Evoked potentials (EP)	Surface electrodes	0.2– 5 k	0.1–20 *μ*	Response of brain potential to stimulus
Local Field Potential (LFP)	Micro electrodes	0.2–200	<5m	Extracellular space in brain tissue
Electromyography (EMG)	Intramuscular and surface electrodes	0.01 to 10 k	0–10 m	Neuromuscular activities

In order to adequately amplify these biological signals of low amplitude, it is necessary to use appropriate amplification techniques while simultaneously mitigating the presence of noise [2]. Three primary strategies employed to eliminate low-frequency components include loading the recording site with a high-value resistor, using active feedback for low-frequency suppression, and utilizing a capacitive feedback network [1]. Analog circuits that utilize transistors functioning in the subthreshold region exhibit reduced energy consumption during active operation and lower leakage power dissipation compared to those operating in the strong inversion zone. The performance attained in the subthreshold area is deemed sufficient for many energy-constrained applications and similar scenarios. Therefore, it is necessary to develop amplifiers specifically designed for biomedical applications that can effectively attain high time constants and high gain. The measurement of brain signals in portable bio-medical monitoring systems is significantly affected by different interfering signals, including electrode offset, flicker noise (1/f), and powerline interference. Hence, to ensure optimal performance, it is imperative to limit the power consumption of the gadget [3].

The design of low-power systems is an essential undertaking that necessitates the constant development of innovative solutions in order to meet the expectations of the industry. In the given environment, the operational transconductance amplifier (OTA) retains its significance as a pivotal component owing to its adaptable nature across a diverse range of applications. Additionally, OTAs possess high-performance characteristics including overall transconductance (Gm), gain-bandwidth product (GBW), slew rate (SR), settling time (ST), area, input-output swing, and noise [4] and they can work within the limitations of low voltage and low power.

This work presents “flipped voltage follower (FVF)” as a potential solution to address some limitations seen in the field [7–9]. Previous studies have demonstrated the utilization of several iterations of this particular cell to achieve low-voltage and low-power functioning. This research delves into an category of Class-AB OTA (Operational Transconductance Amplifier) circuit referred to as variable-mirror amplifier (VMA), highlighting their pivotal role in the development of chopper amplifier. These VMAs are constructed using single-stage topologies that use non-linear current mirrors. However, this paper enhances the concept by incorporating several additional features. These include an extension to the weak inversion operation, the introduction of a new auxiliary biasing circuit to enhance control over peak currents, the derivation of analytical expressions for the maximum slew rate, the provision of a comprehensive set of parametrized curves to demonstrate the design flexibility of the VMA topology, and a thorough analysis of settling time to optimize power consumption in switched-capacitor (SC) circuits [10].

The utilization of the suggested VMA design ensures that Class-AB dynamic peak currents are exclusively directed toward the output transistors. In addition to its inherent power-saving benefits, this single-stage design method effectively circumvents the current and area overheads often associated with frequency compensation techniques. Furthermore, it has the advantage of accommodating load-capacitance capabilities spanning several decades and is compatible with the quick on-off operation in SC circuits. Finally, it is worth noting that the suggested VMA family has a notable degree of technological and temperature insensitivity [11].

The input-referred noise of the amplifier can be mitigated by adjusting the size of the input devices or employing circuit methods, such as chopper stabilization, to minimize the impact of flicker noise. In the context of an action potential sensing application, chopper modulation is implemented at elevated frequencies. Therefore, to maintain the input impedance of the amplifier, it is necessary to perform chopper modulation of the input signal at the virtual ground of the amplifier [12]. The noise experienced at the recording location will be amplified as a result of the reduced input impedance of the amplifier, which is caused by the loading of the electrode impedance. In practical applications, it is important to ensure that the overall input-referred noise of the amplifier remains lower than both the extracellular neuronal background noise and the background noise originating from the electrodes.

In this study, a pseudo-resistors (*R*_*P*_) [1] with resistance values in the range of hundreds of *G*Ω is designed. This design takes into account the issue of leakage via the reverse-biased pn junctions, which has a detrimental effect on the performance of the *R*_*P*_s.

This research incorporates a cutting-edge design featuring a superclass AB OTA [13]. The design includes self-cascode current mirrors (SCCM) and an adaptive biasing approach based on an FVF. The presented OTA is modified to operate with low voltage and low power consumption, aiming to enhance slew rate, DC gain, gain-bandwidth product, and CMRR while minimizing static power dissipation.

The present paper is structured in the following manner: The chopper amplifier section provides an overview of the proposed architectural design and offers a comprehensive analysis of the significant parameters of the amplifier. The implementation and design of the high-gain amplifier are presented in the OTA circuit section. The next section presents a pseudo-resistor and chopper modulation technique for noise analysis and reduction. The results section comprises various simulation results performed using Cadence Virtuoso. Finally, concluding remarks of the work is presented as separate section.

## Architecture of chopper amplifier with adaptive biasing technique

**Fig 1:**
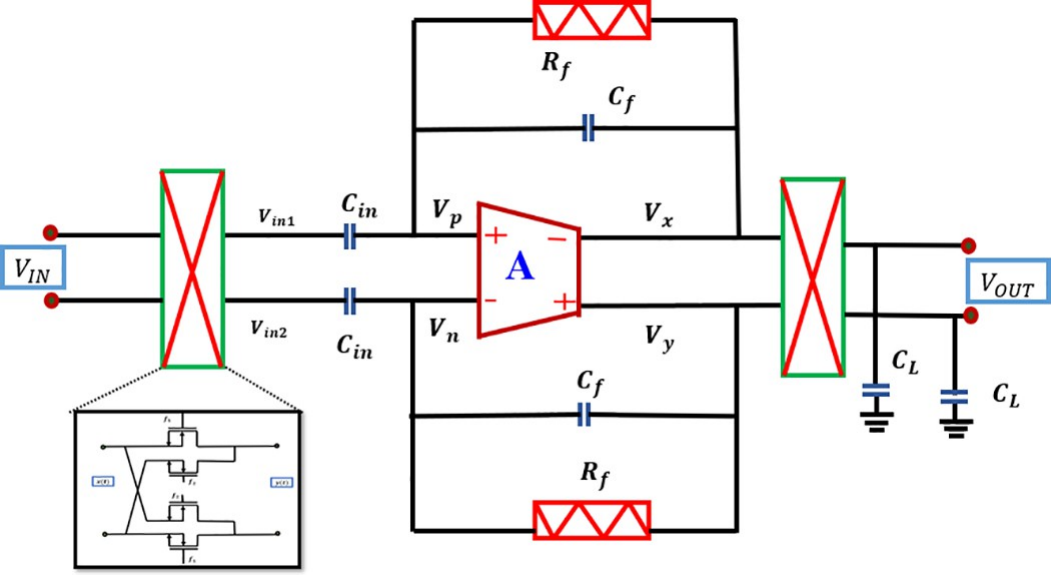
Architecture of the switched capacitor amplifier using chopper modulator.

The proposed system architecture is depicted in Fig 1. It comprises a low noise chopper modulator, a single-stage suggested OTA with FVF topology denoted as ‘A’, and a capacitive feedback loop utilized for establishing the mid-band gain. Additionally, *R*_*P*_s are employed to provide substantially increased resistance.

The chopper modulation technique aims to improve the CMRR of the differential amplifier and mitigate the presence of flicker noise in the CMOS transistors. The input chopper, without altering the common-mode signal, enables the core amplifier to treat it as a standard input. The basic amplifier converts the common input into differential output by the utilization of the common mode gain. However, the differential mode output voltage is altered by the output modulator due to the non-zero gain of the common mode amplifier. As a result, it is possible to remove it by the same process used for filtering out flicker (1/f) noise and input offset voltage [14].

Traditionally, AC-coupled amplifiers are implemented by including a DC-blocking capacitor in series at the input. However, the attainment of extremely low cut-off frequencies sometimes necessitates the use of quite large capacitors. This is a drawback in bio-potential recording applications because of the substantial silicon area occupied by these capacitors. The impedance seen at the input of a current mode chopper-stabilized amplifier may be approximated as 1/(2*πf*_*chop*_*C*_*in*_), where *f*_*chop*_ represents the chopping frequency occurs and *C*_*in*_ denotes the DC-blocking capacitor connected in series [15, 16].

A feasible alternative to active-RC integrator is the utilization of MOSFETs functioning within the linear operational range as a replacement for resistors. In this particular scenario, the MOSFETs are commonly referred to as *R*_*P*_s. In the context of weak inversion, it has been observed that *R*_*P*_s can exhibit a significant bias-induced effect, leading to the manifestation of remarkably high channel resistances. The resistances *R*_*P*_s, in conjunction with integrated capacitors, can attain the notably extensive time constants necessary for applications involving very low frequencies. However, it is important to note that achieving this requires the utilization of exceedingly low bias currents [17].

It is evident that best possible performance, it is imperative to employ a completely differential amplifier that incorporates symmetrical feedback. However, it is possible to efficiently regulate the gain by using just one feedback line. The analytical circuit shown in Fig 1 achieves balanced feedback paths by using matching resistors, labeled as *R*_*f*_.

The closed-loop transfer function of the amplifier [18] can be expressed as:

$$\begin{array}{c} \frac{V_{\text{out}\;}}{V_{\text{in}}}=[1+\frac{WLC_{ox}}{C_{in}}{]}^{2}A_{M}\frac{1}{1+\frac{1}{LG(s)}} \end{array}$$

$$\begin{array}{c} =[1+\frac{WLC_{ox}}{C_{in}}{]}^{2}[\frac{C_{\text{in}\;}}{C_{f}}\frac{sR_{f}C_{f}}{\left(1+sR_{f}C_{f}\right)}\times \frac{1}{\left(1+\frac{1+sR_{f}\left(C_{f}+C_{in}\right)}{G_{m}r_{o}\left(1+sR_{f}C_{f}\right)}\right)}]. \end{array}$$ (1)

The comprehensive breakdown of the aforementioned equations can be found in Appendix A. The *C*_*in*_/*C*_*f*_ ratio is a quantitative metric that may be employed to determine the mid-band gain, denoted as (*A*_*M*_) and *G*_*m*_ represents the transconductance. The lower cutoff frequency may be determined by the formula 1/(*R*_*f*_*C*_*f*_), where *R*_*f*_ represents the feedback resistance and *C*_*f*_ represents the feedback capacitance. The analysis shown above does not consider the electrode resistance (*R*_*s*_) because of its negligible impact compared to the feedback component (*R*_*f*_). The contribution of noise from the *R*_*f*_ and OTA to amplifiers is significantly reduced as a result of the extremely narrow bandwidth [19].

The FVF-OTA rejects the input and output common-mode voltages, preventing them from entering. Similarly, the output voltage is dictated by the offset voltage. The circuit being discussed utilizes asymmetrical feedback and utilizes offset voltage to affect Vout uniquely, resulting in the presence of offset voltage in output. The deficiency of symmetry in the differential amplifier causes imbalances in the operating points of its internal nodes, resulting in a reduction in the alignment of open-loop gains. Although CMRR may not be a major issue with single-ended inputs, the research highlights a considerable trade-off in CMRR when nonsymmetrical feedback is used.

By leveraging the aspect ratio correlations outlined in Table 2, we can derive the amplifier specifications necessary for our requirements. When designing a bio-potential amplifier, it is essential to consider the trade-offs between noise and power consumption. The selection of device size is a deliberate process aimed at optimizing amplifier performance metrics, including input-referred noise, low-frequency gain, input impedance, and bandwidth, with the ultimate goal of achieving the optimum amount of channel inversion.

**Table 2:** Aspect ratio of architecture.

Components	W/L (*μm*)	Components	W/L (*μm*)
M1,M2	45/3	M3,M4,M5,M6,M7,M8	20/1
Ma6,Ma7	3/6	*MP*_1_,*MP*_2_,*MP*_3_,*MP*_4_	6/3
Ma5,Ma8	6/6	*MP*_5_,*MP*_6_*MP*_7_,*MP*_8_	6/3
Ma3,Ma4	15/6	MC1,MC2,MC3,MC4	6/3
M1A,M1B	20/1	M2A,M2B	20/1

### Circuit architecture

**Fig 2:**
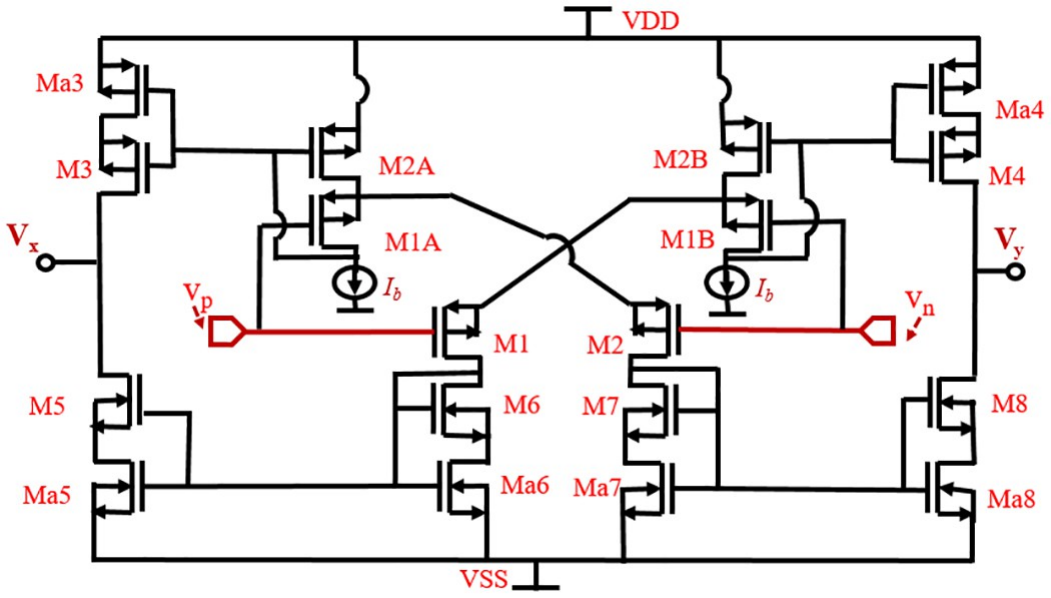
Schematic of FVF-OTA used in amplifier [13].

The implementation of a class-AB OTA based on the FVF adaptive biasing circuit (ABC) technique is depicted in Fig 2. The ABC consists of a pair of input differential transistors, M1 and M2, which are matched and cross-coupled by the two DC-level shifters. The implementation of each DC level shifter involves the utilization of two FVFs [13], which are comprised of transistors M1A, M2A, M1B, M2B, and a current source *I*_*b*_. The active loads in this system are realized through the use of SCCM architecture. The driver transistors, namely Ma3–Ma8, are biased close to the linear region. On the other hand, the cascode transistors, denoted as M3–M8, operate either in the linear region or the saturation region, depending on the voltage bias applied to their gates. The transition occurring between the linear and saturation regions of the transistors results in the intended amplification of current [13, 20, 21].

The traditional class A OTA can evolve into a superclass AB OTA through the replacement of the constant bias current source (*I*_*b*_) with an ABC. The SCCM architecture has been implemented utilizing a class AB PMOS differential pair. The ABC allows for a significantly higher current to be delivered compared to the quiescent current when a large differential input signal is present. This circuit is specifically engineered to provide minimal quiescent currents to transistors M1 and M2, while automatically boosting the bias current when substantial differential input signals are detected. To elevate current amplification and optimize output swing, the input differential pair is replaced with SCCMs [22].

The class AB configuration in OTA, demonstrates significantly increased current output when exposed to a substantial differential input voltage, exceeding its quiescent currents. The tail is bifurcated into two transistors (M1 and M2), each linked to the source terminal of the differential pair and the source degeneration active load. Positioned between the source terminals of the differential pair is a symmetrically configured source degeneration active load comprised of transistors M3 and M4. Importantly, this load is individually forward-body-biased through the buffered input voltage signals *V*_*p*_ and *V*_*n*_ [5, 23].

The gain of the suggested OTA is determined by analyzing the analog low-frequency signals. In the depicted diagram, *V*_*p*_ and *V*_*n*_ denote the absolute values of the input voltages that are applied at the gate terminals of MOSFETs M1 and M2, respectively. $g_{m_{K}}$ represents the transconductance of the *K*_*th*_ MOSFET, where K equals to 1, 2, 1A, 2A, 1B, and 2B.

$$\begin{array}{c} {\text{g}}_{{\text{m}}_{\text{K}}}=\sqrt{{\text{I}}_{{\text{B}}_{\text{K}}}\mu_{\text{k}}{\text{C}}_{\alpha \text{x}}[\frac{\text{W}}{\text{L}}{]}_{\text{K}}} \end{array}$$ (2)

In the given context, *μ*_*K*_ represents the carrier mobility, W/L is the transistor aspect ratio, and $I_{B_{K}}$ represents the bias current of the *K*_*th*_ transistor [13]. The disregard of channel length modulation in *K*_*th*_ transistors is done to simplify the mathematical formulas.

$$\begin{array}{c} A_{V_{dm}}=G_{m}r_{o}=(g_{m_{1}}+g_{m_{2}})(r_{{\text{o}}_{{\text{eff}}_{4}}}\|r_{{\text{o}}_{{\text{eff}}_{8}}}) \end{array}$$ (3)

The overall gain of the OTA denoted as $A_{V_{dm}}$ and detailed analysis of the above equations is available in Appendix B. The variables $g_{m_{k}}$ and $r_{o_{eff_{P}}}$ represent the effective transconductance and effective output resistance, respectively, of *P*_*th*_ self-cascode transistors (MP and MaP). The value of P can range from 3–8. In order to enhance the range of maximum output, the output transistors M4 and M8 are interconnected in a conventional common-source design. Under quiescent circumstances, the transistors M5, M6, M7, and M8 function within the subthreshold region.

### Pseudo-resistor

**Fig 3:**
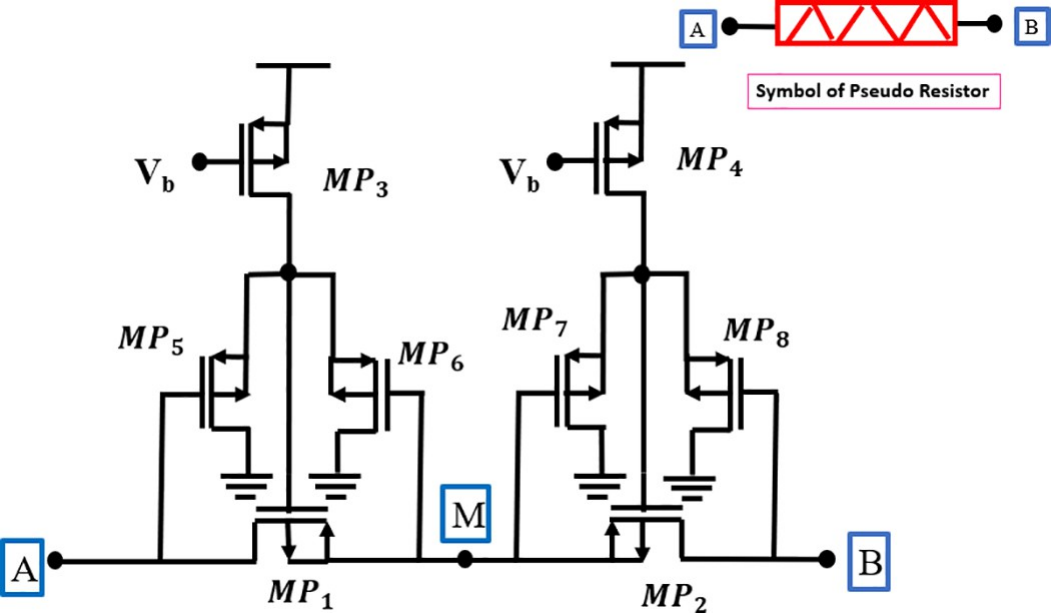
MOSFET based psuedo resistor [3].

The fundamental unit of the floating pseudo-resistor (*R*_*P*_) in Fig 3 utilizes transistors *MP*_1_ and *MP*_2_ to function as a resistor. These transistors are connected in series to augment the overall resistance. The transistors *MP*_3_ and *MP*_8_ are utilized as the biasing circuit with a current source *I*_*b*_. The achievement of higher resistance values, denoted as *R*_*P*_, at low cutoff frequencies. These resistors exhibit a notable advantage in terms of occupying less physical space compared to passive resistors. It is worth noting that previous *R*_*P*_ values were biased at the subthreshold inversion point. The voltage follower, specifically the *MP*_1_–*MP*_2_ configuration, serves to transform a voltage that is referred to ground into a voltage reference that is floating [1].

In the present work, the resistance value attained with the topology is described when the *R*_*P*_ operates in the subthreshold region.

$$\begin{array}{c} {\left.R_{P}\right|}_{V_{AB}=0}=\frac{2\phi_{t}}{I_{b}}\frac{{\left(W/L\right)}_{MP_{1}}}{{\left(W/L\right)}_{MP_{2}}}. \end{array}$$ (4)

The symbol *ϕ*_*t*_ represents the thermal voltage, and the aspect ratios W/L of the transistors *MP*_1_ and *MP*_2_, respectively. Based on the findings, it is evident that there exist two distinct design techniques that might be employed to get a significant value of *R*_*P*_. Initially, it is imperative to minimize the bias current, which can be accomplished by using a very precise current reference and/or employing down-mirroring techniques. Additionally, the ratio between the aspect ratios of *MP*_1_ and *MP*_2_ must be significant [24].

The adjustment of the aspect ratios necessary for the design was achieved by the utilization of a combination of series connections of transistors. To enhance resistance and linearity, a series connection was established between *R*_*P*_s biased by distinct voltage followers. It should be noted that the *R*_*P*_’s segments are biased using a sole current source (*I*_*b*_) and a single reference transistor (*MP*_1_). Furthermore, it is unnecessary to assign individual *I*_*b*_ and *MP*_1_ to each segment of the *R*_*P*_, as demonstrated in reference, since the floating voltage $V_{MP_{1}}$ remains constant throughout all segments [25, 26].

*R*_*P*_s are subject to significant constraints that stem from parasitic effects, including the direct current (DC) leakage and the capacitance of reverse-biased pn-junctions, along with the dispersed capacitance of the transistor channel.

### Low noise chopper modulator

**Fig 4:**
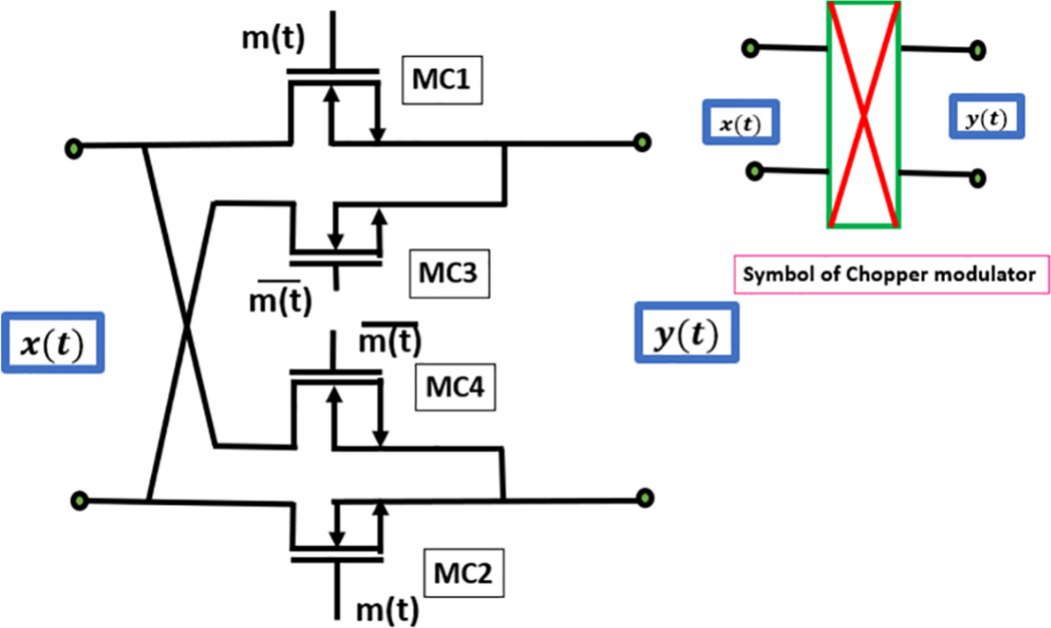
Chopper amplifier input modulator circuit diagram [27].

The block diagram depicted in Fig 4 illustrates the chopper circuit, which is readily built with MOS technology and it requires only four switches MC1, MC2, MC3, and MC4 for a complete differential chopper implementation. The passive chopper circuits directly take the input signal from the electrode and are considered to be noise-critical. The process of achieving signal sign inversion involves the straightforward alternation of the signal route by using a clock and its counter-phase. The utilization of the clock counter phase in dummy switches enables the cancellation of the main switch charge to a first-order approximation through the absorption and release of the dummy MOSFET charge [27].

In the field of CMOS technology, the utilization of MOSFETs offers a straightforward approach to implementing switches. The input chopper transforms a DC signal input into a square wave. The process of chopping entails the utilization of two polarity-reversing choppers to achieve accurate modulation and demodulation. The chopper configuration of four switches controlled by clock signals exhibiting two complementary phases, operating at a certain chopping frequency denoted as *f*_*chop*_. Following amplification, the output chopper performs demodulation on the square wave signal, resulting in the restoration of DC voltage. In the context of frequency analysis, the input chopper efficiently shifts the DC signal to the odd harmonics of the chopping frequency, and then the output chopper restores the high-frequency components to the DC level [28].

Typically, the act of chopping does not result in the introduction of additional noise, particularly when the choppers are situated at low-impedance nodes. In the given scenario, the primary source of noise may be attributed to the on-resistance of the input chopper. Therefore, by reducing this value to a sufficiently low level, the noise generated by it is rendered inconsequential.

## Noise analysis of the chopper amplifier

**Fig 5:**
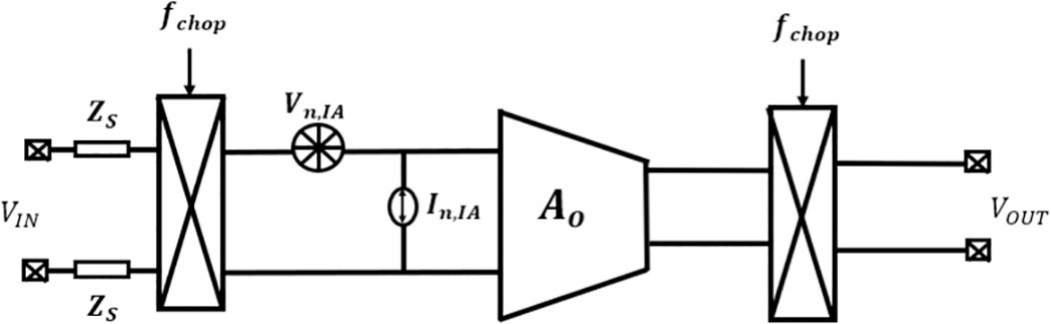
Noise model of the amplifier.

Fig 5 illustrates the noise model of a chopper amplifier. The representation of the amplifier with noise may be equivalently expressed as a voltage source (*V*_*n*,*IA*_) in series with a noiseless amplifier and a current source (*I*_*n*,*IA*_) in parallel with the same noiseless amplifier [29, 30].

In the realm of designing low-noise amplifiers in CMOS technology, the primary sources of noise are commonly attributed to thermal noise and 1/f noise. This section will conduct a theoretical analysis of the impact of chopping modulation on the noise performance of an amplifier [30, 31]. The total input-referred noise power spectral density (PSD) is given by

$$\begin{array}{c} S_{N,in}\left(f\right)=\bar{V_{n,IA}^{2}}+2\bar{I_{n,IA}^{2}}Z_{s}^{2}. \end{array}$$ (5)

$$\begin{array}{c} S_{N,in}\left(f\right)=S_{N0}(1+\frac{f_{c}}{f}). \end{array}$$ (6)

where *Z*_*S*_ is the impedance of the source. In the case of low frequencies and high input impedance, the dominant term in Eq 5 is the first term. The rearrangement of equation Eq 5 results in the addition of a thermal noise term, denoted as *S*_*N*0_, together with a low-frequency *1/f* noise term as shown in Eq 6, where *f*_*c*_ represents the corner frequency. Subsequently, the input noise of comparable magnitude is amplified by the voltage gain, denoted as *A*_0_, and subjected to modulation through the output chopper. The PSD of the output noise is obtained by summing the copies of the noise spectrum that are positioned at odd harmonic frequencies of the chopping frequency (*f*_*chop*_) [30, 32, 33].

$$\begin{array}{c} \begin{array}{c} S_{N,\;\text{out}\;}\left(f\right)=(\frac{2}{\pi }{)}^{2}\sum_{k=-\infty }^{+\infty }\frac{1}{k^{2}}A_{0}^{2}S_{N,\;\text{in}\;}(f-kf_{\text{chop}\;}).\\ k=1,3,5,\ldots \end{array} \end{array}$$ (7)

$$\begin{array}{c} \begin{array}{c} S_{N,\;\text{out}\;,\;\text{baseband}\;}\left(f\right)≅A_{0}^{2}S_{N0}(1+\frac{17f_{c}}{2\pi^{2}f_{\text{chop}}})\\ (\;\text{for}\;f_{\text{cutoff}\;}\gg f_{\text{chop}\;}). \end{array} \end{array}$$ (8)

The variable “*f*_*cutoff*_” represents the cut-off frequency of the amplifier. It is evident from Eq 8 that the noise level at the baseband is essentially equivalent to the thermal noise, *S*_*N*0_ when the amplifier’s bandwidth is significantly greater than the chopping frequency.

## Simulation results

**Fig 6:**
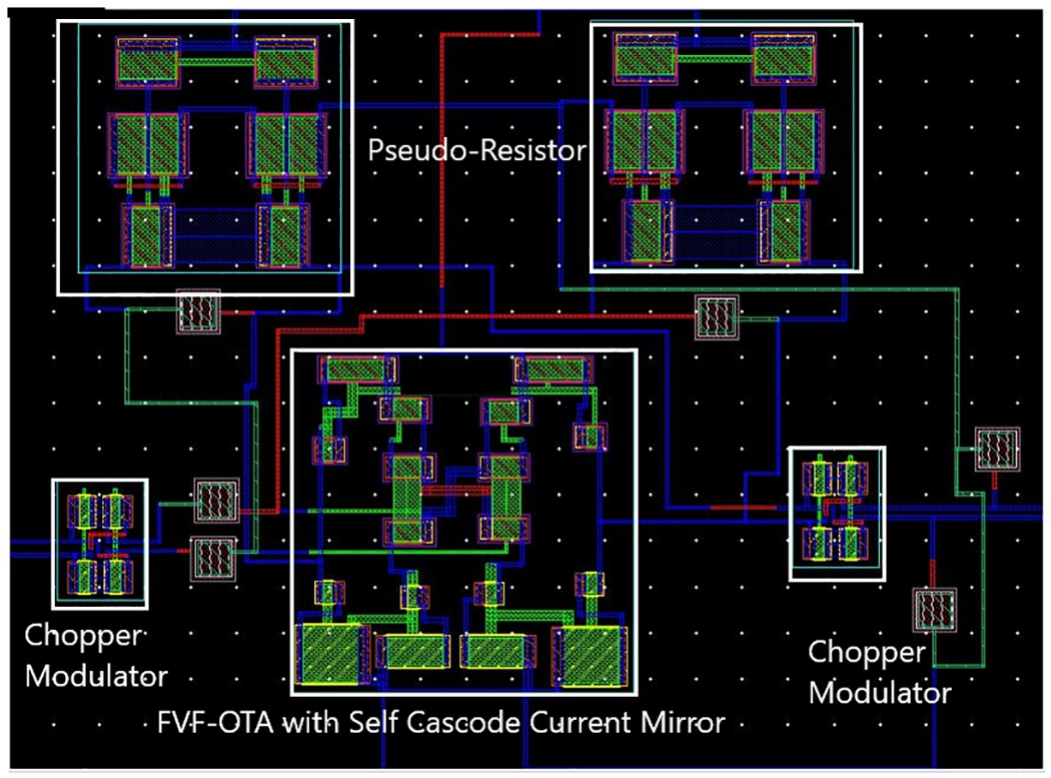
Layout of the proposed circuit.

The evaluation and performance of the proposed switched capacitor chopper amplifier based on FVF-OTA design using a 180 nm CMOS technology node, was conducted with a chopping frequency of 30 kHz. The biopotential amplifier presented utilizes the FVF-OTA and employs a cascode current mirror to generate the gain and CMRR. The amplifier achieves a gain of 63.72 dB, effectively amplifying it within the frequency range of 0.1 Hz to 12.17 kHz. The circuit functions by utilizing 29.61 *μW* of power, while exhibiting an IRN of 0.07 *nV*_*rms*_. In depicted Fig 6 a simulated capacitive feedback amplifier has a layout design size of 0.009 *mm*^2^. The layout characteristics are achieved through the utilization of MIM-based capacitors.

**Fig 7:**
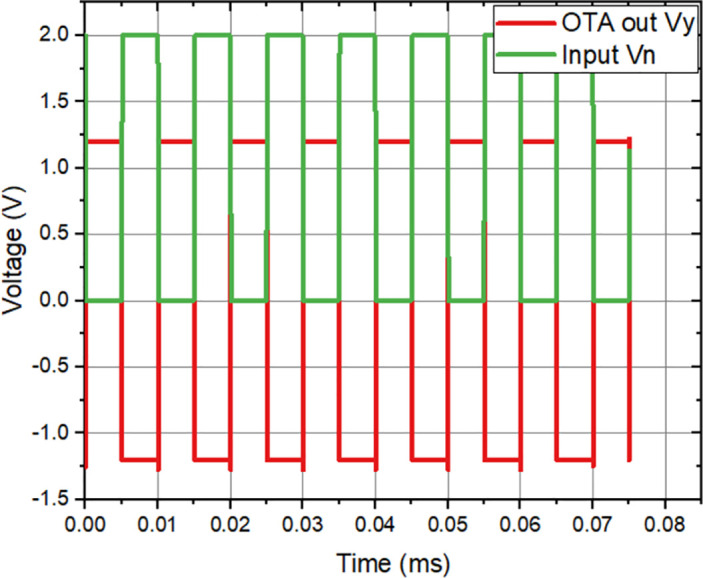
Simulated time-domain analysis of the OTA.

The amplifier that was developed with input capacitance (*C*_*in*_) of 3 *μ*F, feedback capacitance (*C*_*f*_) of 1 pF, and load capacitance (*C*_*L*_) of 3 pF. To imitate the functioning of the circuit, a sinusoidal signal is employed. This signal possesses an amplitude of 100 mV, a frequency of 100 Hz, and a DC offset of 50 mV, which is introduced to compensate for the electrode offset voltage. In order to analyze the behavior during startup and transient phases, it is necessary to replicate the transient response of the circuit across a reasonable time range, as depicted in Fig 7. The significance of Class-AB operation in low-frequency amplifiers is of utmost relevance owing to its capacity to offer both efficiency and linearity. Class-AB amplifiers are characterized in that each amplifying device operates for a duration slightly exceeding half of the input signal cycle. Their suitability for low-frequency applications require precise signal reproduction due to their ability to minimize crossover distortion. Additionally, it delves into the compromises involved in choosing the most suitable biasing point for class-AB operation, taking into account the balance between quiescent current and distortion performance.

**Fig 8:**
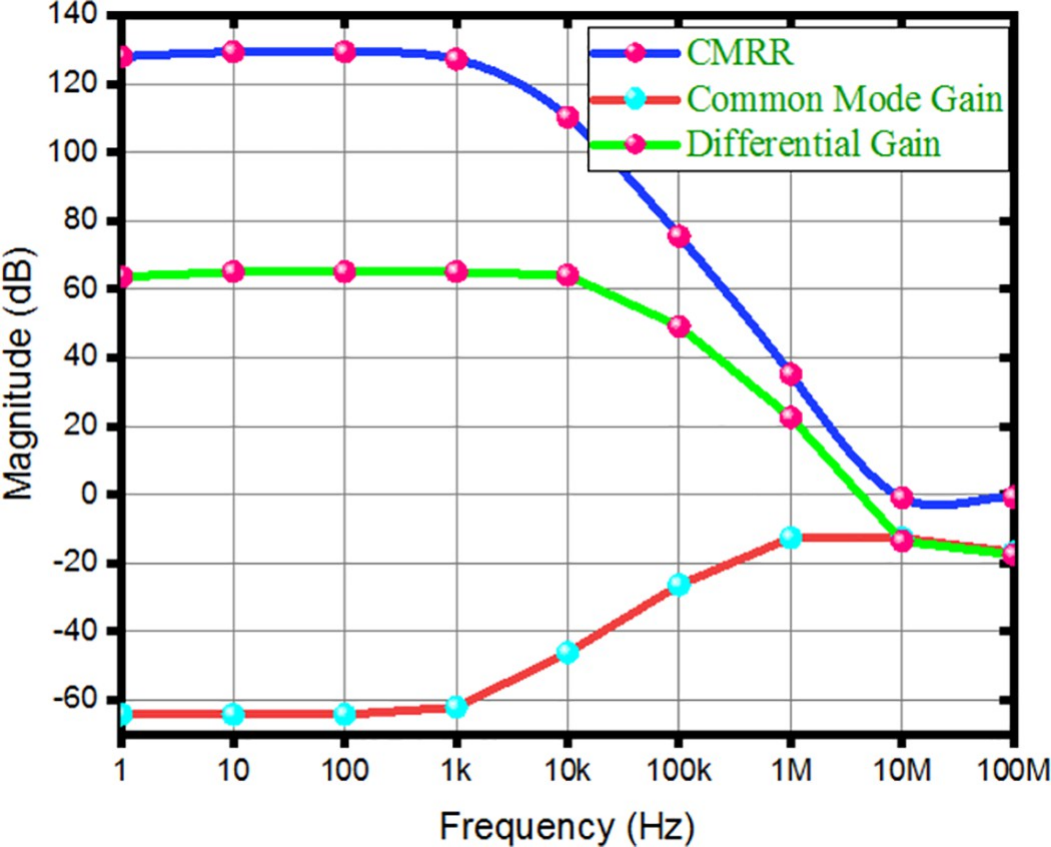
Simulated CMRR, differential gain, and common mode gain of the proposed amplifier.

Differential gain and CMRR indicate the amplifier’s effectiveness in rejecting common-mode signals. Incorporating the FVF adaptive biasing technique into the chopper amplifier makes it easier to maintain a steady state of operation. Consequently, CMRR and differential gain improved by limiting bias current fluctuations and reducing common-mode distortion. The closed-loop differential gain, common-mode gain, CMRR are measured to be 63.72 dB, -64.25 dB, and 127.97 dB respectively, as depicted in Fig 8. When the gain decreases by 3 dB, the frequency range spans from 0.1 Hz to 12.17 kHz, and the bandwidth is constrained.

**Fig 9:**
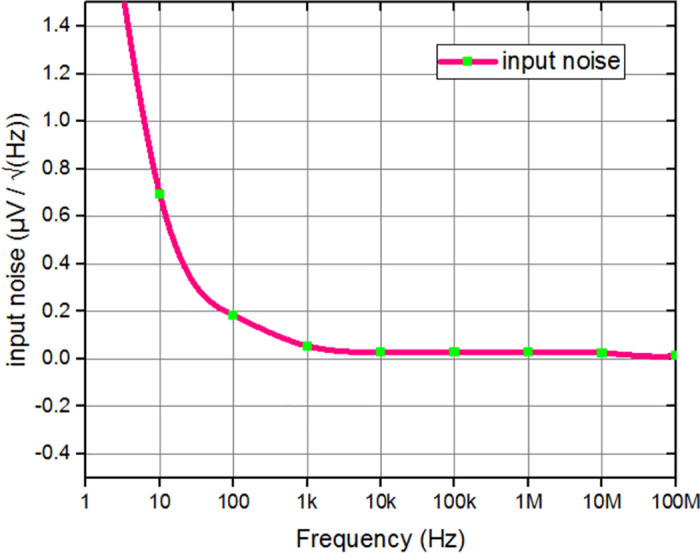
Input noise analysis of proposed circuit.

**Fig 10:**
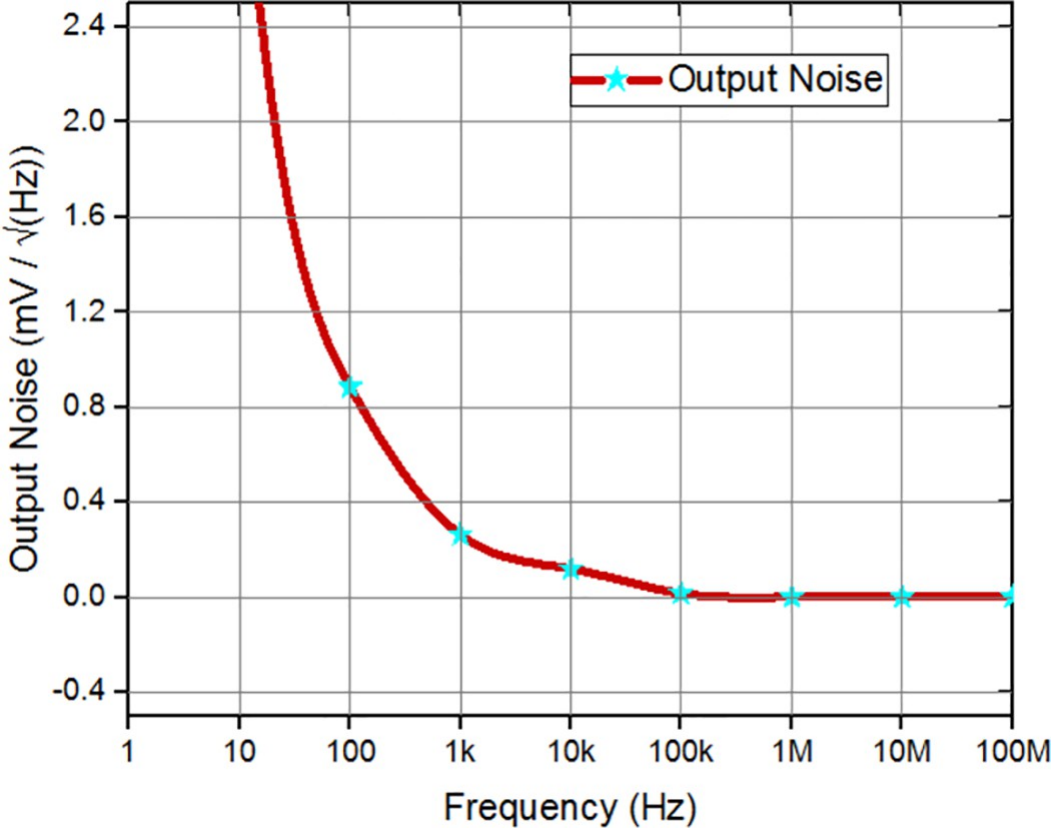
Output noise analysis of proposed circuit.

The proposed architecture was employed to amplify and process biological signals, which are inherently of small amplitude. The reduction of flicker noise generated by the adaptive biasing of the FVF can be accomplished by employing filtering methods, such as the utilization of a low-pass filter or the application of noise cancelation techniques. The utilization of feedback control techniques in this context enables the stabilization of the amplifier performance and enhances its resilience against noise induced by the FVF adaptive biasing circuitry. Hence, the investigation of noise has been carried out by the utilization of simulation techniques. The measurements of input noise and output noise were obtained at levels of 159.847 $nV/\sqrt{Hz}$ and 0.77 $mV^{2}/\sqrt{Hz}$, respectively, as depicted in Figs 9 and 10. The presented data illustrates the noise characteristics of has been improved for the circuit design under consideration.

The chopping technique does mitigates *1/f* noise effectively, which is its primary advantage. However, the process introduces elements (switched capacitor resistance and high-value capacitors) that can increase input impedance *Z*_*in*_. Therefore, while it might seem that chopping should reduce the input impedance, the introduction of these components and their associated resistances actually results in an increase in the overall input impedance. As a result, the impedance in the FVF-OTA architecture typically exceeds 1.545 *G*Ω. Therefore, it has been implemented to minimize loading effects and increase input impedance to achieve the desired gain and CMRR performance.

**Fig 11:**
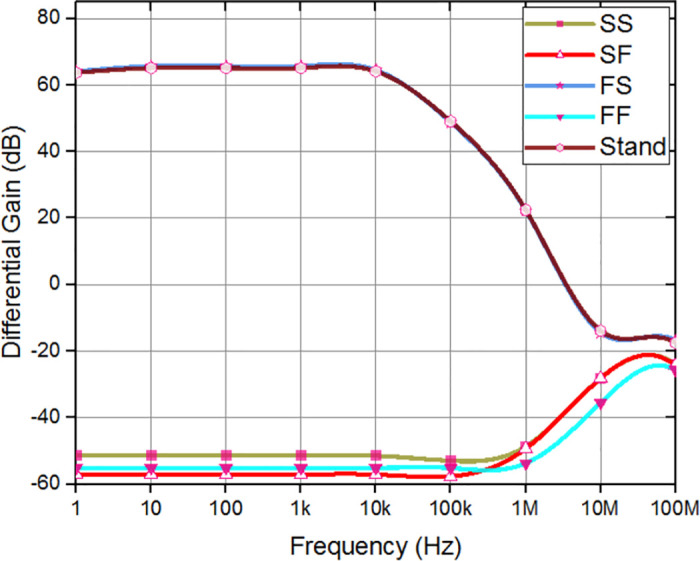
Differential gain corner analysis of overall design.

**Fig 12:**
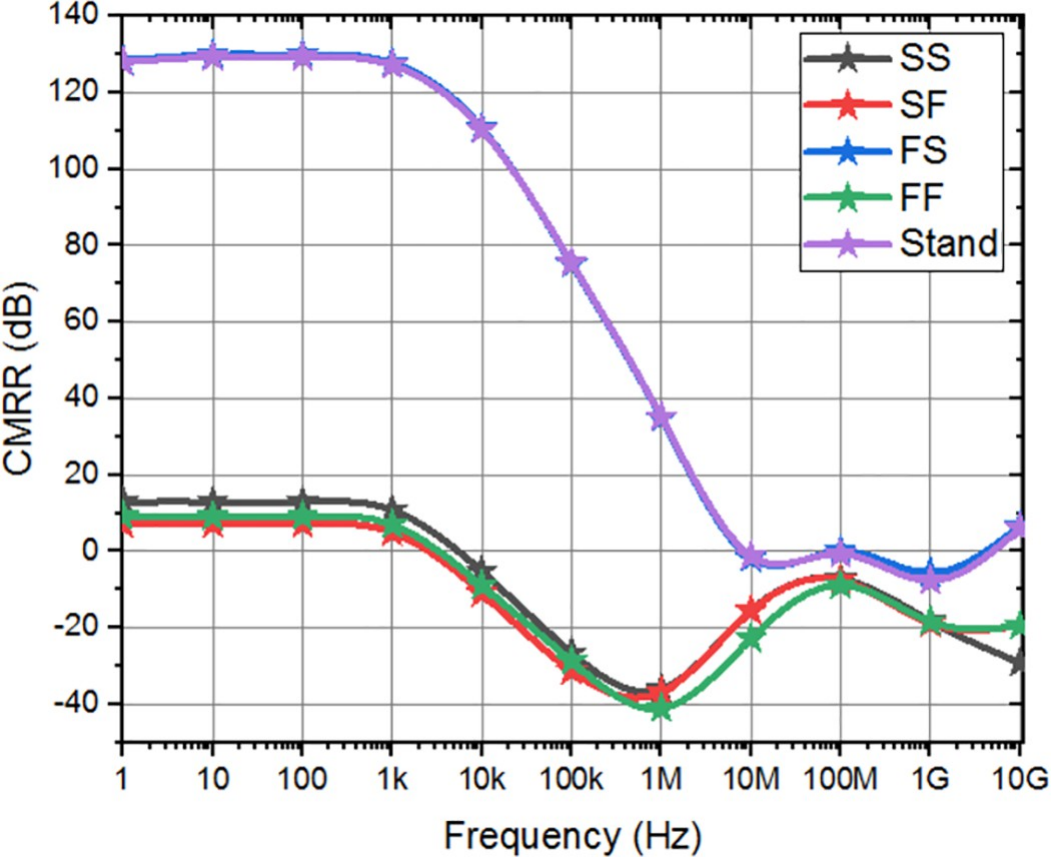
CMRR corner analysis of overall design.

**Fig 13:**
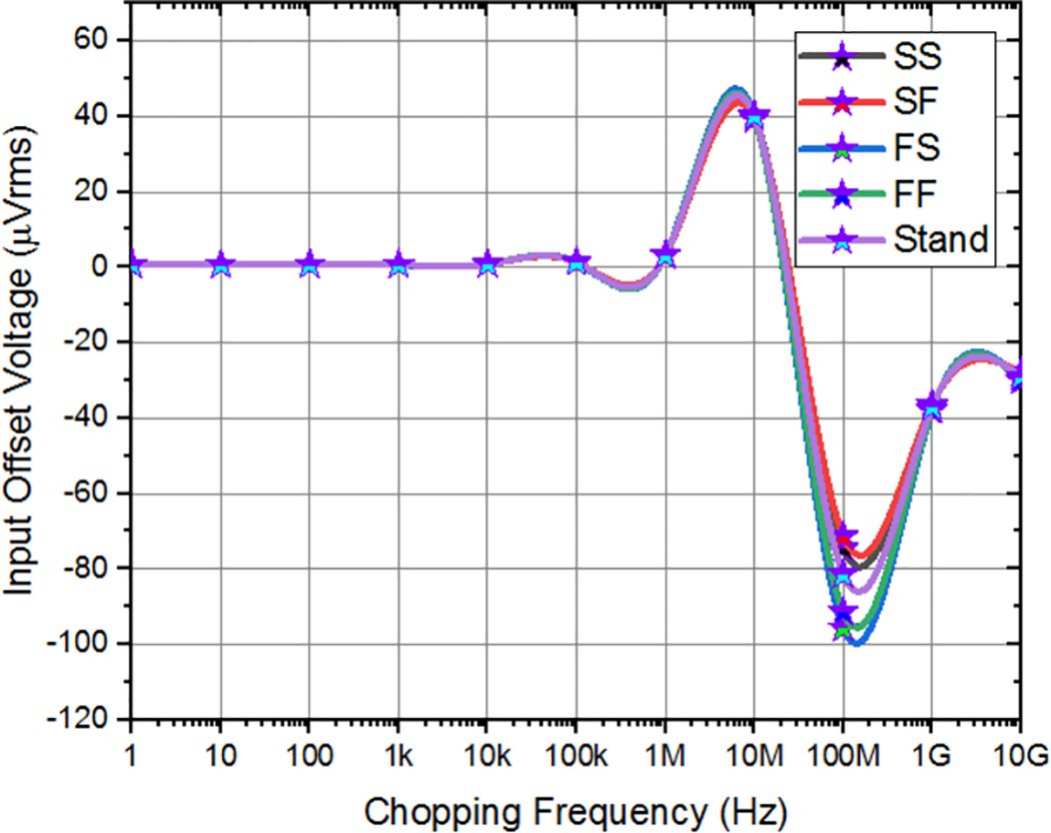
Input-offset voltage corner analysis of amplifier.

Corner analysis involves simulations under different supply voltage corners to assess the amplifier’s response to supply voltage variations. It serves as a crucial tool to assess the effectiveness of adaptive amplifiers in preserving intended performance metrics under worst-case scenarios by consistently demonstrating performance in process, temperature, and supply voltage corners. The differential gain, and CMRR of the amplifier indicate a little deviation from the anticipated level, and various corners have been performed as shown in Figs 11 and 12, respectively. As depicted in the Fig 13, the offset voltage demonstrates a progressive rise throughout the frequency range of 1 MHz to 30 MHz, which can be attributed to common interference. Subsequently, it stabilizes within a gradual range once again. In order to comprehensively depict the behavior of offset voltage across the whole frequency spectrum of interest, it is imperative to incorporate a suitably broad frequency range within the plot. It will guarantee the most efficient frequency range for chopping, according to individual applications and performance criteria. The designed amplifier performance observes that Stand and FS corner frequencies exhibit somewhat sophisticated values compared to the simulated values.

Table 3 illustrates the performance metrics of the amplifier under examination, along with comparisons to findings from related studies. This comparison reveals that the suggested amplifier outperforms others in gain, CMRR, and exhibits notably high *Z*_*in*_. The minimum attainable supply voltage for the recommended amplifier is primarily constrained by the elevated threshold voltages inherent in the transistors utilized within the 180 nm CMOS technology, aligning with trends observed in comparable amplifiers presented. Furthermore, the power dissipation is relatively significant, reaching a maximum value of 29.61 *μW*.

**Table 3:** The proposed circuit’s comparison table with the outcomes of prior implementations.

Work	[34]	[35]	[36]	[37]	[20]	[38]	[39]	This work
Technology (*μ*m)	**0.18**	**0.18**	0.13	0.18	0.18	**0.18**	**0.18**	**0.18**
Supply Voltage(v)	1.8	1.2	2	1.8	1.2	1.2	1.8	**0.8**
Current(*μA*)	16.1	5.8	2.52	16.1	2	3.7	1.8	**3.7 4.9**
Gain(dB)	58.1	58	43–55	58.1	39.2	40	40	**63.72**
CMRR					78	94	>100	**127.97**
Chopping Frequency (kHz)		20	20	20	20			**30**
Power Dissipation (*μW*)		3.65	35.8	4.4	4.3	4.4	3.24/Channel	**29.61**
Bandwidth (Hz)	170–9680	0.5–500	40–320	170—9.68 k	0.25—2 k			**0.5–12.17k**
Input Impedance (Ω)	> 60	> 200				17 M	440 M	**1.545G**
Integrated Input referred Noise (*μV*_*rms*_)	3.21	3.83	3.54	5.79(1 Hz-10 kHz)	3.9	0.65(0.3–200Hz) 2.14(0.2k-5kHz)		**0.07 (1 Hz-10 kHz)**

**Fig 14:**
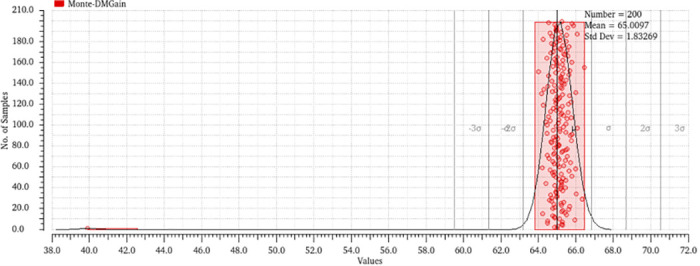
Monte-Carlo analysis of differential mode gain output.

**Fig 15:**
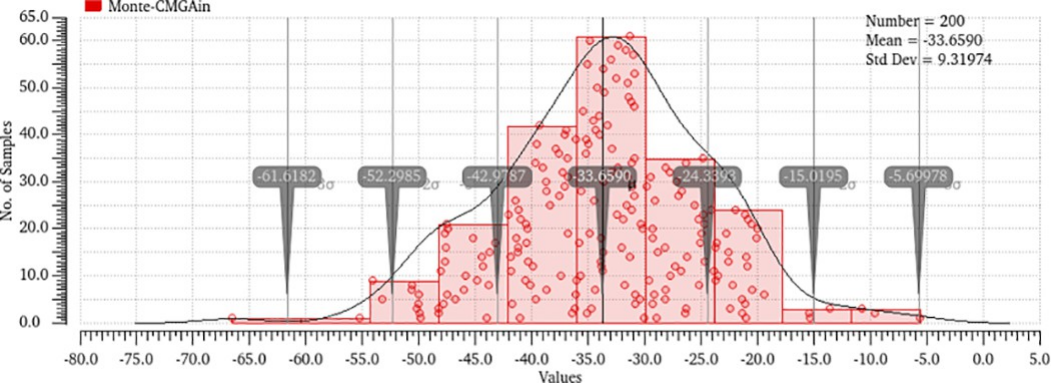
Monte-Carlo analysis of common mode gain output.

**Fig 16:**
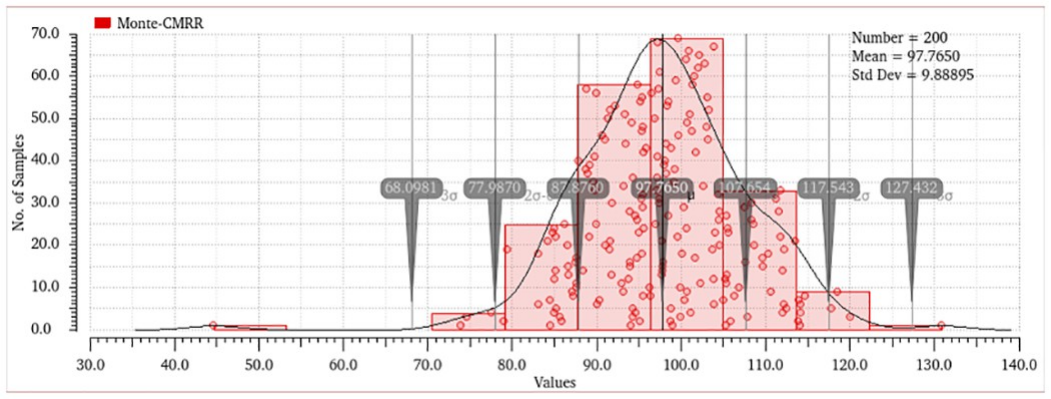
Monte-Carlo Analysis of system CMRR.

**Fig 17:**
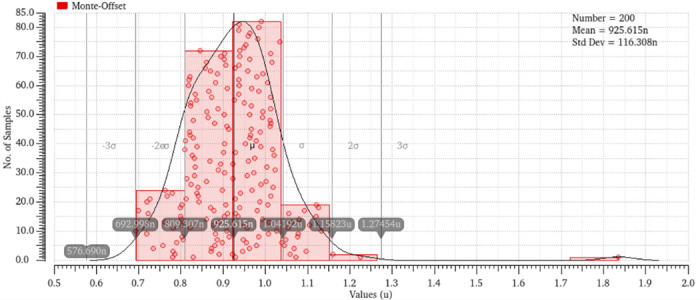
Monte-Carlo analysis of system offset voltage.

Monte Carlo simulations offer a statistical examination of amplifier performance in the presence of process fluctuations, enabling designers to evaluate the efficacy of adaptive approaches in mitigating the standard deviation of performance measurements. This evaluation involved the utilization of 200 Monte Carlo simulations, which considered the effects of mismatches and various factors. The histogram representing the differential gain, common mode gain, and CMRR may be observed in Figs 14–17. The bio-potential amplifier exhibits minor variations for the majority of these parameters. For instance, the mean values of the differential gain, CMRR, common mode gain, and offset voltage are 65.009 dB, 97.76 dB, -33.65, and 925.615 nv respectively, and the standard deviations are 1.83, 9.88, 9.31, and 116.308 nV, respectively. The observed decrease in standard deviation suggests that the amplifier design exhibits enhanced robustness and durability when employed in real-world scenarios. Evaluation of the robustness and reliability of an amplifier design considers not only the reduction in standard deviation but also other key performance metrics. Furthermore, it showcases their ability to maintain consistent performance benchmarks amidst diverse and challenging conditions, encompassing fluctuations in processes, temperature shifts, and fluctuations in supply voltage.

**Fig 18:**
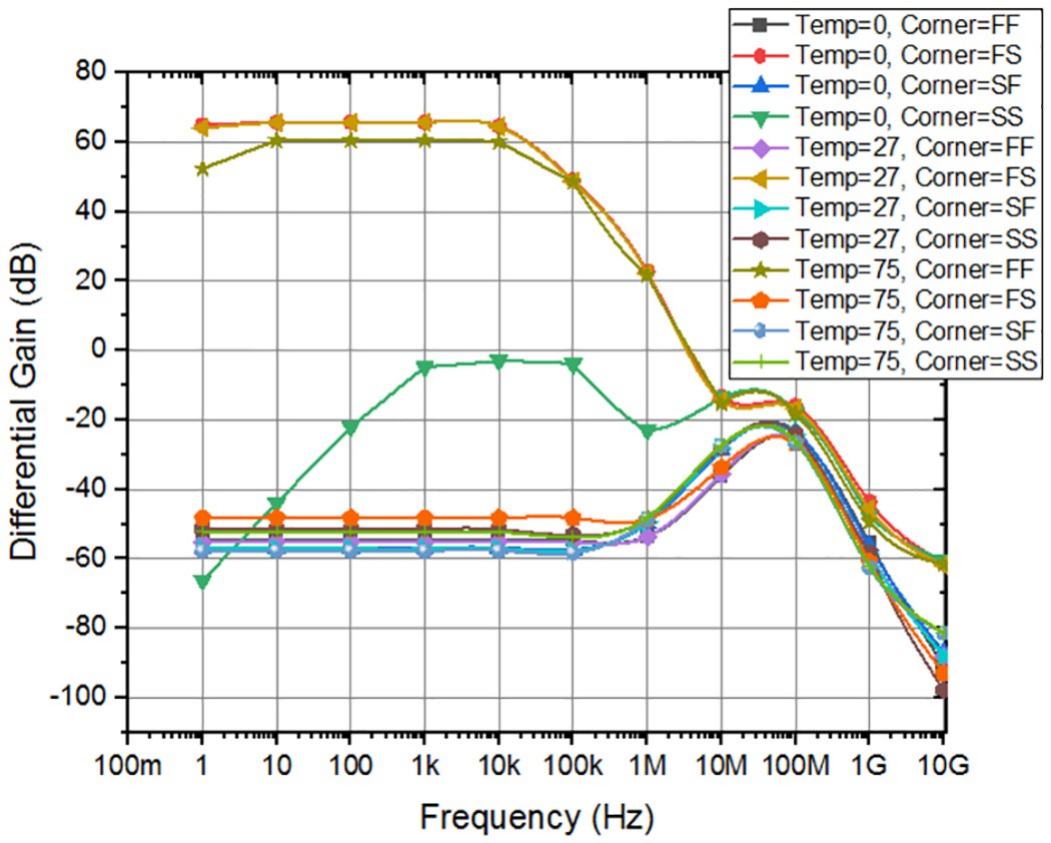
Differential gain with different temperatures and supply for the amplifier.

The results of the voltage and temperature variation analysis are illustrated in Fig 18. A comprehensive investigation of the circuit’s performance under challenging conditions of Corner fluctuations and temperature changes was conducted. To ensure precision across various temperature ranges (0°*C*, 27°*C*, 75°*C*) and corner limits (FF FS SF SS), the system underwent thorough scrutiny for fluctuations in both gain and CMRR, all kept within a 10% margin. This scrutiny serves as compelling evidence of the system’s accuracy and performance. The presented evidence is crucial in validating the efficacy of adaptive amplifier designs in real-world scenarios, as it showcases their capacity to achieve the intended performance even in the most unfavorable conditions.

## Conclusion

This work presents a high-performance switched capacitor chopper amplifier based on the FVF adaptive biasing technique and SCCMs-based active load. This modified amplifier is an excellent alternative where the input signal is significantly weaker than the common mode signal, such as those found in bio-potential amplifiers. The suggested chopper amplifier design demonstrates a notable improvement in gain and CMRR, which are 63.72 dB and 127.97 dB, respectively. The utilization of the suggested amplifier increases the input impedance while simultaneously achieving low noise and low power consumption. The simulation results of the suggested amplifier are analyzed using the Cadence Virtuoso tool, employing a 180 nm CMOS technology node and a supply voltage of 0.8 V. These findings are subsequently compared to pertinent results in the current literature and validated through Monte Carlo simulations exhibiting reduced standard deviation. Ultimately, it has been shown that the CMRR can be improved by introducing capacitive load in alternative SC amplifiers, provided that it does not impact the differential gain. Overall, the suggested work provides an enabling technology for biological applications.

## A Appendix

By applying the superposition theorem to the loop circuit of Fig 1 at node “p” loop equation is

$$\begin{array}{c} \begin{array}{c} V_{p}=v_{in1}(\frac{R_{f}\|C_{f}}{\frac{1}{SC_{in}}+(R_{f}\|C_{f})})+v_{x}(\frac{\frac{1}{SC_{in}}}{\frac{1}{SC_{in}}+(R_{f}\|C_{f})}). \end{array} \end{array}$$ (9)

Here feedback loop is

$$\begin{array}{c} \begin{array}{c} (R_{f}\|C_{f})=(\frac{R_{f}}{1+SR_{f}C_{f}}). \end{array} \end{array}$$

Let

$$\begin{array}{c} \begin{array}{c} \beta =\frac{\frac{1}{SC_{in}}}{\frac{1}{SC_{in}}+(R_{f}\|C_{f})}=\frac{1+{\text{SR}}_{\text{f}}C_{f}}{1+SR_{f}(C_{f}+C_{in})}. \end{array} \end{array}$$

$$\begin{array}{c} 1-\beta =1-\frac{1+{\text{sR}}_{\text{f}}C_{f}}{1+{\text{SR}}_{\text{f}}(C_{f}+C_{in})}=\frac{{\text{SR}}_{\text{f}}C_{in}}{1+SR_{f}(C_{f}+C_{in})}. \end{array}$$

Now

$$\begin{array}{c} V_{p}=v_{in1}(\frac{SR_{f}C_{in}}{1+SR_{f}(C_{f}+C_{in})})+V_{x}(\frac{1+SR_{f}C_{f}}{1+SR_{f}(C_{f}+C_{in})}). \end{array}$$ (10)

$$\begin{array}{c} V_{p}=v_{in1}\left(1-\beta \right)+V_{x}\left(\beta \right). \end{array}$$ (11)

In the same way node “n” loop equation is

$$\begin{array}{c} V_{n}=v_{in2}\left(1-\beta \right)+V_{y}\left(\beta \right). \end{array}$$ (12)

The differential amplifier input-output relationship is

$$(V_{x}-V_{y})=A_{F}(V_{n}-V_{p}).$$

$$\begin{array}{c} (V_{x}-V_{y})=A_{F}[(v_{in2}\left(1-\beta \right)+V_{y}\left(\beta \right))-(v_{in1}\left(1-\beta \right)+V_{x}\left(\beta \right)]. \end{array}$$ (13)

$$\begin{array}{c} \begin{array}{c} (V_{x}-V_{y})=\frac{A_{F}\left(1-\beta \right)}{1+A_{F}\beta }(v_{in2}-v_{\text{in1}}). \end{array} \end{array}$$ (14)

The closed-loop gain transfer function across the amplifier without chopper modulators is

$$\begin{array}{c} \frac{\left(V_{x}-V_{y}\right)}{\left(v_{in2}-v_{\text{in1}}\right)}=\frac{\left(1-\beta \right)}{\beta }(\frac{1}{1+(\frac{1}{A_{F}\beta })}), \end{array}$$

$$\begin{array}{c} =(\frac{c_{in}}{c_{f}})(\frac{{\text{SR}}_{\text{f}}{\text{C}}_{\text{f}}}{1+SR_{f}C_{f}})(\frac{1}{1+(\frac{1+SR_{f}(C_{f}+C_{in}}{G_{m}r_{o}\left(1+SR_{f}C_{f}\right)})}). \end{array}$$ (15)

Assume *A*_*F*_*β* ≫ 1 then

$$\begin{array}{c} \frac{\left(V_{x}-V_{y}\right)}{\left(v_{in2}-v_{\text{in1}}\right)}=\frac{\left(1-\beta \right)}{\beta }=\frac{{\text{SR}}_{\text{f}}{\text{C}}_{\text{in}}}{1+SR_{f}C_{f}}. \end{array}$$ (16)

## B Appendix

The OTA analysis

$$\begin{array}{c} \begin{array}{c} {\text{V}}_{{\text{gs}}_{1A}}=({\text{V}}_{\text{n}}-{\text{V}}_{{\text{D}}_{2A}}),{\text{V}}_{{\text{gs}}_{2A}}={\text{V}}_{{\text{D}}_{1A}}. \end{array} \end{array}$$ (17)

$$\begin{array}{c} \begin{array}{c} \;{\text{V}}_{{\text{gs}}_{1B}}=({\text{V}}_{\text{p}}-{\text{V}}_{{\text{D}}_{2B}}),{\text{V}}_{{\text{gs}}_{2B}}={\text{V}}_{{\text{D}}_{1B}}. \end{array} \end{array}$$ (18)

$$\begin{array}{c} \begin{array}{c} \;{\text{V}}_{{\text{gs}}_{1}}={\text{V}}_{\text{n}}-{\text{V}}_{{\text{D}}_{2B}}. \end{array} \end{array}$$ (19)

$$\begin{array}{c} \begin{array}{c} \;{\text{V}}_{{\text{gs}}_{2}}={\text{V}}_{\text{p}}-{\text{V}}_{{\text{D}}_{2A}}. \end{array} \end{array}$$ (20)

Apply KCL at different nodes in FVF-OTA

$$\begin{array}{c} {\text{g}}_{{\text{m}}_{{\text{eff}}_{6}}}{\text{V}}_{{\text{D}}_{1}}+\frac{{\text{V}}_{{\text{D}}_{1}}}{{\text{r}}_{{\text{o}}_{{\text{eff}}_{6}}}}={\text{g}}_{{\text{m}}_{1}}\;{\text{V}}_{{\text{gs}}_{1}}. \end{array}$$ (21)

$$\begin{array}{c} \begin{array}{c} \;{\text{g}}_{{\text{m}}_{{\text{eff}}_{7}}}{\text{V}}_{{\text{D}}_{2}}+\frac{{\text{V}}_{{\text{D}}_{2}}}{{\text{r}}_{{\text{e}}_{{\text{eff}}_{7}}}}={\text{g}}_{{\text{m}}_{2}}\;{\text{V}}_{{\text{gs}}_{2}}. \end{array} \end{array}$$ (22)

$$\begin{array}{c} \begin{array}{c} \;{\text{V}}_{{\text{D}}_{1A}}={\text{R}}_{{\text{Bg}}_{{\text{m}}_{1A}}}\;{\text{V}}_{{\text{gs}}_{1A}}. \end{array} \end{array}$$ (23)

$$\begin{array}{c} \begin{array}{c} \;{\text{V}}_{{\text{D}}_{1B}}={\text{R}}_{{\text{Bg}}_{{\text{m}}_{1B}}}\;{\text{V}}_{{\text{gs}}_{1\text{B}}}. \end{array} \end{array}$$ (24)

$$\begin{array}{c} \begin{array}{c} {\text{g}}_{{\text{m}}_{1A}}\;{\text{V}}_{{\text{gs}}_{1A}}+{\text{g}}_{{\text{m}}_{2}}\;{\text{V}}_{{\text{gs}}_{2}}={\text{g}}_{{\text{m}}_{2A}}\;{\text{V}}_{{\text{gs}}_{2A}}. \end{array} \end{array}$$ (25)

$$\begin{array}{c} \begin{array}{c} \;{\text{g}}_{{\text{m}}_{1B}}\;{\text{V}}_{{\text{gs}}_{1B}}+{\text{g}}_{{\text{m}}_{1}}\;{\text{V}}_{{\text{gs}}_{1}}={\text{g}}_{{\text{m}}_{2B}}\;{\text{V}}_{{\text{gs}}_{2B}}. \end{array} \end{array}$$ (26)

From (17) and (23); (assume *R*_*B*_ ≫ 1), $V_{D_{2A}}$ can be simplified as

$${\text{V}}_{{\text{D}}_{2A}}\approx {\text{V}}_{\text{n}}.$$

Similarly, from (18) and (24) (assume *R*_*B*_ ≫ 1), $V_{D_{2B}}$ can be simplified as

$${\text{V}}_{{\text{D}}_{2B}}\approx {\text{V}}_{\text{p}}.$$

$$\begin{array}{c} \;{\text{g}}_{{\text{m}}_{{\text{eff}}_{3}}}{\text{V}}_{{\text{D}}_{3}}+{\text{g}}_{{\text{m}}_{{\text{eff}}_{5}}}{\text{V}}_{{\text{D}}_{1}}+\frac{{\text{V}}_{{\text{D}}_{3}}}{{\text{r}}_{{\text{o}}_{{\text{eff}}_{3}}}}+\frac{{\text{V}}_{{\text{D}}_{3}}}{{\text{r}}_{{\text{o}}_{{\text{eff}}_{5}}}}+I_{on}=0. \end{array}$$ (27)

$$\begin{array}{c} \;{\text{g}}_{{\text{m}}_{\text{eff}4}}{\text{V}}_{{\text{D}}_{3}}+{\text{g}}_{{\text{m}}_{\text{eff}8}}{\text{V}}_{{\text{D}}_{2}}+\frac{{\text{V}}_{{\text{D}}_{8}}}{{\text{r}}_{{\text{o}}_{{\text{eff}}_{4}}}}+\frac{{\text{V}}_{{\text{D}}_{8}}}{{\text{r}}_{{\text{o}}_{{\text{eff}}_{8}}}}+I_{op}=0. \end{array}$$ (28)

From Eq (27), (assume $r_{o_{{\text{eff}}_{3}}}$ and $r_{o_{{\text{eff}}_{5}}}\gg 1$), the value of voltage *I*_*on*_ can be written as

$${\text{I}}_{\text{on}}=-{\text{g}}_{{\text{m}}_{{\text{eff}}_{3}}}{\text{V}}_{{\text{D}}_{3}}-{\text{g}}_{{\text{m}}_{{\text{eff}}_{5}}}{\text{V}}_{{\text{D}}_{1}}.$$

From Eq (28), (assume $r_{o_{{\text{eff}}_{4}}}$ and $r_{o_{{\text{eff}}_{8}}}\gg 1$), the output current *I*_*op*_ can be written as

$${\text{I}}_{\text{op}}=-{\text{g}}_{{\text{m}}_{{\text{eff}}_{4}}}{\text{V}}_{{\text{D}}_{3}}-{\text{g}}_{{\text{m}}_{{\text{eff}}_{8}}}{\text{V}}_{{\text{D}}_{2}}.$$

the output current *I*_*o*_ can be written as

$$\begin{array}{c} {\text{I}}_{\text{o}}=I_{op}-I_{on}=-{\text{g}}_{{\text{m}}_{{\text{eff}}_{8}}}{\text{V}}_{{\text{D}}_{2}}+{\text{g}}_{{\text{m}}_{{\text{eff}}_{5}}}{\text{V}}_{{\text{D}}_{1}}. \end{array}$$ (29)

Using Eqs (21) and (22) (assuming ${\text{g}}_{{\text{m}}_{{\text{eff}}_{3}}}={\text{g}}_{{\text{m}}_{{\text{eff}}_{4}}},{\text{g}}_{{\text{m}}_{{\text{eff}}_{5}}}={\text{g}}_{{\text{m}}_{{\text{eff}}_{6}}},{\text{g}}_{{\text{m}}_{{\text{eff}}_{7}}}={\text{g}}_{{\text{m}}_{{\text{eff}}_{8}}},{\text{r}}_{{\text{o}}_{{\text{eff}}_{6}}}$ and ${\text{r}}_{{\text{o}}_{{\text{eff}}_{7}}}\gg 1$),

*I*_*o*_ can be written as

$$\begin{array}{c} {\text{I}}_{\text{o}}=I_{op}-I_{on}=-{\text{g}}_{{\text{m}}_{2}}{\text{V}}_{{\text{gs}}_{2}}+{\text{g}}_{{\text{m}}_{1}}{\text{V}}_{{\text{gs}}_{1}}. \end{array}$$ (30)

Value of $V_{{\text{gs}}_{1}}$ and ${\text{V}}_{{\text{gs}}_{2}}$ can be obtained from Eqs (19) and (20). Therefore, G_m_ can be obtained as

$$\begin{array}{c} {\text{G}}_{\text{m}}=\frac{{\text{I}}_{\text{o}}}{{\text{V}}_{\text{p}}-{\text{V}}_{\text{n}}}=-({\text{g}}_{{\text{m}}_{1}}+{\text{g}}_{{\text{m}}_{2}}). \end{array}$$ (31)

The output resistance (r_O_) of OTA is given as

$$\begin{array}{c} {\text{r}}_{\text{o}}={\text{r}}_{{\text{o}}_{{\text{eff}}_{4}}}\|{\text{r}}_{{\text{o}}_{{\text{eff}}_{8}}}. \end{array}$$ (32)

Using Eqs (31) and (32) the gain can be expressed as

$$\begin{array}{c} {\text{A}}_{\text{F}}=\frac{{\text{V}}_{o}}{\left(\;{\text{V}}_{\text{p}}-{\text{V}}_{\text{n}}\right)}=-({\text{g}}_{{\text{m}}_{1}}+{\text{g}}_{{\text{m}}_{2}})({\text{r}}_{{\text{o}}_{{\text{eff}}_{4}}}\|{\text{r}}_{{\text{o}}_{{\text{eff}}_{8}}}). \end{array}$$ (33)

## Supporting information

S1 Data (ZIP)

## References

[1] Ansari F, Yavari M. A Fully-Differential Chopper Capacitively-Coupled Amplifier with High Input Impedance for Closed-Loop Neural Recording. Circuits, Systems, and Signal Processing. 2022;41(7):3679–3705. doi: 10.1007/s00034-022-01970-3

[2] Arce-Zavala J, Vazquez AM, Padilla-Cantoya I, Plascencia F. A Gm-C Notch Filter Implemented With g m Over I D Technique For Biosignal Acquisition Systems. In: 2020 17th International Conference on Electrical Engineering, Computing Science and Automatic Control (CCE). IEEE; 2020. p. 1–6.

[3] Ansari M, et al. Analog front-end design for biomedical signal acquisition systems. CSI Transactions on ICT. 2019;7(3):199–204. doi: 10.1007/s40012-019-00232-z

[4] Chandrakumar H, Marković D. An 80-mVpp Linear-Input Range, 1.6- G Ω Input Impedance, Low-Power Chopper Amplifier for Closed-Loop Neural Recording That Is Tolerant to 650-mVpp Common-Mode Interference. IEEE Journal of Solid-State Circuits. 2017;52(11):2811–2828.

[5] Harrison RR, Charles C. A low-power low-noise CMOS amplifier for neural recording applications. IEEE Journal of solid-state circuits. 2003;38(6):958–965. doi: 10.1109/JSSC.2003.811979

[6] Renuga M, Gnanambal K. Low power high performance CMOS amplifier for epileptic seizure prediction. Analog Integrated Circuits and Signal Processing. 2021;109(2):387–402. doi: 10.1007/s10470-021-01918-8

[7] Ramirez-Angulo J, Baswa S, López-Martín AJ, Carvajal RG. Winner-take-all class AB input stage: A novel concept for low-voltage power-efficient class AB amplifiers. In: 2004 IEEE International Symposium on Circuits and Systems (IEEE Cat. No. 04CH37512). vol. 1. IEEE; 2004. p. I–1028.

[8] Garde MP, Lopez-Martin A, Ramírez-Angulo J. Enhanced differential super class-AB OTA. In: 2017 13th Conference on Ph. D. Research in Microelectronics and Electronics (PRIME). IEEE; 2017. p. 137–140.

[9] Carvajal RG, Ramírez-Angulo J, López-Martín AJ, Torralba A, Galán JAG, Carlosena A, et al. The flipped voltage follower: A useful cell for low-voltage low-power circuit design. IEEE Transactions on Circuits and Systems I: Regular Papers. 2005;52(7):1276–1291. doi: 10.1109/TCSI.2005.851387

[10] Dabas A, Gupta M, Yadav R, Kumari S. Design and Analysis of High-Performance Double Recycling Folded Cascode Operational Transconductance Amplifier. Iranian Journal of Science and Technology, Transactions of Electrical Engineering. 2023; p. 1–19.

[11] Sutula S, Dei M, Terés L, Serra-Graells F. Variable-mirror amplifier: A new family of process-independent class-AB single-stage OTAs for low-power SC circuits. IEEE Transactions on Circuits and Systems I: Regular Papers. 2016;63(8):1101–1110. doi: 10.1109/TCSI.2016.2577838

[12] Li Y, Poon CC, Zhang YT. Analog integrated circuits design for processing physiological signals. IEEE Reviews in Biomedical Engineering. 2010;3:93–105. doi: 10.1109/RBME.2010.2082521 22275203

[13] Mahendra M, Kumari S, Gupta M, Sangal A. Low voltage high performance super class AB OTA design using SCCM and DTMOS with enhanced slew rate and DC gain. Microelectronics Journal. 2021;113:105101. doi: 10.1016/j.mejo.2021.105101

[14] Zhang J, Zhang H, Sun Q, Zhang R. A low-noise, low-power amplifier with current-reused OTA for ECG recordings. IEEE transactions on biomedical circuits and systems. 2018;12(3):700–708. doi: 10.1109/TBCAS.2018.2819207 29877832

[15] Yin M, Ghovanloo M. A low-noise preamplifier with adjustable gain and bandwidth for biopotential recording applications. In: 2007 IEEE International Symposium on Circuits and Systems. IEEE; 2007. p. 321–324.

[16] Cao W, Liu Y, Liu S, Wang L, Ma R, Zhu Z. A 2.6 GΩ, 1.4 *μ*Vrms current-reuse instrumentation amplifier for wearable electrocardiogram monitoring. Microelectronics Journal. 2021;107:104940. doi: 10.1016/j.mejo.2020.104940

[17] Kim HS, Cha HK. A low-noise biopotential CMOS amplifier IC using low-power two-stage OTA for neural recording applications. Journal of Circuits, Systems and Computers. 2018;27(05):1850068. doi: 10.1142/S0218126618500688

[18] Nagulapalli R, Hayatleh K, Barker S, Zourob S, Yassine N. An OTA gain enhancement technique for low power biomedical applications. Analog Integrated Circuits and Signal Processing. 2018;95(3):387–394. doi: 10.1007/s10470-018-1148-y

[19] Sanjay R, Senthil Rajan V, Venkataramani B. A low-power low-noise and high swing biopotential amplifier in 0.18 *μ*m CMOS. Analog Integrated Circuits and Signal Processing. 2018;96(3):565–576. doi: 10.1007/s10470-018-1207-4

[20] Kumar A, Tripathi SL, Verma C, Raboaca MS, Enescu FM, Mihaltan TC. Design and Analysis of Low Power Bio-amplifier with Current Mirror Topology at CMOS 45nm Technology Node. In: 2022 14th International Conference on Electronics, Computers and Artificial Intelligence (ECAI). IEEE; 2022. p. 1–5.

[21] Lopez-Martin A, Garde MP, Carvajal RG, Ramírez-Angulo J. On the optimal current followers for wide-swing current-efficient amplifiers. In: 2018 IEEE International Symposium on Circuits and Systems (ISCAS). IEEE; 2018. p. 1–5.

[22] Bernal MRV, Celma S, Medrano N, Calvo B. An ultralow-power low-voltage class-AB fully differential OpAmp for long-life autonomous portable equipment. IEEE Transactions on Circuits and Systems II: Express Briefs. 2012;59(10):643–647.

[23] Psychalinos C, Yesil A, Minaei S, Bertsias P. Flipped Voltage Follower-Based Voltage Conveyors: Investigation and Possible Enhancements. Circuits, Systems, and Signal Processing. 2023;42(4):2028–2048. doi: 10.1007/s00034-022-02230-0

[24] Rezaee-Dehsorkh H, Ravanshad N, Lotfi R, Mafinezhad K. A linear tunable amplifier for implantable neural recording applications. In: 2011 IEEE 54th International Midwest Symposium on Circuits and Systems (MWSCAS). IEEE; 2011. p. 1–4.

[25] Nagulapalli R, Hayatleh K, Barker S, Georgiou P, Lidgey F. A high value, linear and tunable cmos pseudo-resistor for biomedical applications. Journal of Circuits, Systems and Computers. 2019;28(06):1950096. doi: 10.1142/S0218126619500968

[26] Li MZ, Tang KT. A low-noise low-power amplifier for implantable device for neural signal acquisition. In: 2009 Annual International Conference of the IEEE Engineering in Medicine and Biology Society. IEEE; 2009. p. 3806–3809. 10.1109/IEMBS.2009.5335204 19965237

[27] Yazicioglu RF, Van Hoof C, Puers R. Biopotential readout circuits for portable acquisition systems. Springer Science & Business Media; 2008.

[28] Fan Q, Sebastianen F, Huijsing H, Makinwa K. A 2.1 *μ*W area-efficient capacitively-coupled chopper instrumentation amplifier for ECG applications in 65 nm CMOS. In: 2010 IEEE Asian Solid-State Circuits Conference. IEEE; 2010. p. 1–4.

[29] dos Reis CA. A Review of Offset and Noise Reduction Techniques for CMOS. Journal of Integrated Circuits and Systems. 2022;17(1):1–9. doi: 10.29292/jics.v17i1.572

[30] Huang YK, Rodriguez S. Noise Analysis of Current-Feedback DC-Servo Loop in Current-Balancing Chopper Amplifiers. In: 2022 IEEE Nordic Circuits and Systems Conference (NorCAS). IEEE; 2022. p. 1–6.

[31] Vafaei M, Parhizgar A, Abiri E, Salehi MR. A low power and ultra-high input impedance analog front end based on fully differential difference inverter-based amplifier for biomedical applications. AEU-International Journal of Electronics and Communications. 2021;142:154005.

[32] Nielsen JH, Bruun E. A CMOS low-noise instrumentation amplifier using chopper modulation. Analog Integrated Circuits and Signal Processing. 2004;42(1):65–76. doi: 10.1023/B:ALOG.0000042329.18883.8a

[33] Kasipogula BR, Komanapalli G. A High Gain, High CMRR, Low Noise Bio-Potential amplifier based on Switched Capacitor Feedback Amplifier. Journal of Integrated Circuits and Systems. 2024;19(2):1–9. doi: 10.29292/jics.v19i2.813

[34] Liu J, Allstot DJ. A Chopper-Stabilized Switched-Capacitor Front-End for Peripheral Nervous System Recording. IEEE Transactions on Circuits and Systems I: Regular Papers. 2023;. doi: 10.1109/TCSI.2023.3282555

[35] Zheng J, Ki WH, Tsui CY. A fully integrated analog front end for biopotential signal sensing. IEEE Transactions on Circuits and Systems I: Regular Papers. 2018;65(11):3800–3809. doi: 10.1109/TCSI.2018.2854741

[36] Hsu YP, Liu Z, Hella MM. A- 68 dB THD, 0.6 mm 2 active area biosignal acquisition system with a 40–320 Hz duty-cycle controlled filter. IEEE Transactions on Circuits and Systems I: Regular Papers. 2019;67(1):48–59. doi: 10.1109/TCSI.2019.2943904

[37] Liu J, Allstot DJ. A switched-capacitor closed-loop integration sampling front-end for peripheral nerve recording. In: 2021 IEEE Biomedical Circuits and Systems Conference (BioCAS). IEEE; 2021. p. 1–4.

[38] Shad E, Molinas M, Ytterdal T. A Low-power and Low-noise Multi-purpose Chopper Amplifier with High CMRR and PSRR. In: 2020 42nd Annual International Conference of the IEEE Engineering in Medicine & Biology Society (EMBC). IEEE; 2020. p. 3998–4001. 10.1109/EMBC44109.2020.9176224 33018876

[39] Luo D, Zhang M, Wang Z. A low-noise chopper amplifier designed for multi-channel neural signal acquisition. IEEE Journal of Solid-State Circuits. 2019;54(8):2255–2265. doi: 10.1109/JSSC.2019.2913101

